# Assessment of adaptive behavior in people with intellectual disabilities: Design and development of a new test battery

**DOI:** 10.1016/j.heliyon.2024.e31048

**Published:** 2024-05-10

**Authors:** Alicia Boluarte Carbajal, Gina Chávez-Ventura, Jorge Cueva-Vargas, Angel Zegarra-López

**Affiliations:** a Faculty of Health Sciences. Universidad Cesar Vallejo. Lima, Peru; b Program for Continuing Education-SUBE. Universidad Cesar Vallejo, Trujillo, Peru; c Faculty of Psychology, Universidad de Lima. Lima, Peru

**Keywords:** *Adaptive behavior*, *Intellectual disability*, *Test development*, *Psychometrics*, *Peru*

## Abstract

**Background:**

Adaptive behavior is an important characteristic of people with intellectual disabilities, and it has been associated with a person's performance in social and work contexts. Indeed, adaptive behavior denotes what a person does independently, without help, support, reminders, or prompts. In Peru, available measures of adaptive behavior are commercial; thus, there is a need for an open-access tool to assess the adaptive behavior of people with intellectual disabilities. For this reason, the aim of the study was to design and develop a new Adaptive Behavior Test Battery for people from 13 to 60 years old with intellectual disabilities who have an interest in being part of the economically active population.

**Methods:**

A cross-sectional design was defined, starting with a qualitative approach to designing and constructing the item pool for the test battery. Then, quantitative indexes Aiken's V for content validity and Krippendorff's alpha for inter-observer reliability were estimated, resulting in a first version of the three subscales that comprised the test battery. The initial versions were tested on a sample of 566 persons with intellectual disabilities from two regions of Peru: Lima (Coast) and San Martín (Jungle). The internal structure was analyzed under a factor analysis approach, along with internal consistency measures of reliability. Further analyses of invariance regarding gender, region, and age were carried out.

**Results:**

Three observer subscales were proposed: Daily living activities (11 items), Instrumental skills (4 items), and Communication (9 items). All subscales showed excellent psychometric properties denoted by the Aiken's V coefficient, Krippendorff's alpha, factor analysis, internal consistency analysis, and invariance analyses.

**Conclusion:**

The developed a new Adaptive Behavior Test Battery is a useful tool for the measurement of adaptive behavior and the monitoring of social and labor inclusion programs for people with intellectual disabilities.

## 1 Introduction

Intellectual disability is a condition characterized by limitations that begin at the developmental stage, affecting a person's intellectual functioning and adaptive behavior [1]. Intellectual difficulties include limitations in reasoning, solving problems, planning, thinking in an abstractive way, making decisions, and learning. These limitations were usually diagnosed with standardized intelligence tests [2]; however, intelligence test results are not useful indicators to understand the degree to which a person with intellectual disabilities is able to deal with day-to-day activities [3]. To define the severity of the intellectual disability, it is necessary to identify how it affects the adaptive behavior of a person and its consequences within the social and cultural context [2,4]. In this sense, adaptive behavior can be considered the most relevant criteria to determine the severity of the disability [5], because it allows the person to have an independent life and to function adequately in society.

Interestingly, adaptive behavior and intelligence are independent but moderately related to each other [3,6]. Indeed, they both are influenced by a person's development and the context in which he has lived; therefore, measuring them is necessary. Several studies have reported controversial results regarding the correlation between the adaptive behaviour and intelligence of people with intellectual disabilities. These differences depend on the test, samples, and range of intellectual disabilities deficits. A recent meta-analysis has reported an estimated mean correlation of r = 0.51between adaptive behaviour and intelligence; moreover, this correlation depends on the IQ, thus, the correlation is stronger when the IQ is lower and vice versa [7]. Opposingly, a recent study has shown that higher IQ is not necessarily associated with better functioning in presence of people with neurodevelopmental disorders [8]. Therefore, a rigorous assessment of these constructs is necessary when making a diagnosis and evaluation of intellectual disabilities.

Intelligence remains relatively stable, but adaptive skills with appropriate personalized support could be learned and developed over time; moreover, people with intellectual disabilities are able to learn new adaptive skills throughout life and improve their conditions [9,10]. In fact, persons with intellectual disabilities may have or develop some talents and skills that make them eligible to be part of the work force. Despite the fact that they may not be able to have making-decision positions, some of them can do some jobs even better than people without intellectual disabilities [11].

Functional behavior in people with intellectual disabilities is characterized by three main domains: conceptual (communication and academic), social (social and interpersonal abilities), and practical (independent and everyday life) skills [4,12,13], as empirically confirmed through factor analyses in previous research [14]. That being said, measuring adaptive behavior indicates what a person with an intellectual disability does independently, without help, support, reminders, or prompts [15], in addition to assuming responsibilities in their social context [16] and during their transition towards adulthood [17]. For this reason, adaptive behavior is indeed extremely relevant to be considered as a potential candidate for a job within society.

There are many questionnaires to measure adaptive behavior (Table 1), but most of them come from culturally and economically distinct places, and they usually cover all age ranges. Among them, the Vineland Adaptive Behavior Scales-3 measure the progressive development of a person's social competence from birth to age 90. This instrument is a good choice for the evaluation of adaptive functioning; however, it has highly variable test-retest stability and the items are generic [18]; moreover, its short version is still long with 180 items. Another option is the Adaptive Behavior Scale-School (ABS-S:2), created to be used by people ranging in age from 3 to 21 years old. This instrument is composed of two sections: adaptive behavior and problem behavior [19]; adaptive behavior also involves three domains such as personal self-sufficiency, personal-social responsibility, and community responsibility [14].

**Table 1:** Existing questionnaires for measuring adaptive behaviour.

Test	Origin	Target population	Description	Psychometric evidence	Factorial structure
Vineland Adaptive Behavior Scales, Third Edition [13].	EEUU	From 0 to 90 years old.	- Interview form for parents/caregivers (502 items full version, 180 short version) and teacher form (333 items). - Online or manual application and qualification. - The full versions measure 9 main subdomains and 5 optional.	- Content validity and internal structure - Concurrent validity, with the previous version of the test, and by correlating the scores of different informants. Comparisons were also made according to age, sex, and educational level. The scores of people with intellectual disabilities and developmental delay were also compared with their previous diagnosis. - The internal consistency for the Alpha Coefficient for the comprehensive scale varied between 0.94 and 0.99; and, for the adaptive domains it was between 0.86 and 0.97	- Core domains: Communication, daily living skills and socialization.
AAMR Adaptive Behavior Scales: School (ABS-S:2) [19].	EEUU	From 3 to 21 years Samples: people with and without mental retardation.	- The items are multiple choice and true/false options. - Manual grading. - It contains two areas: personal independence and social maladjustment.	- Content validity. - Only the first part of the test was moderately correlated with Vineland Scale scores. - Adequate Cronbach's Alpha values (0.79–0.98) and stability with the test-retest and inter-observer method.	- Made up of subscales: independence, language development, responsibility, socialization, maladaptive behaviors
Adaptive Behavior Assessment System, third edition (ABAS-3) [20].	EEUU	From birth to 89 years of age. It contains 10 areas corresponding to the domains: conceptual, social and practical. Communication, Community Use, Functional Academics, School Living, Health and Safety, Leisure, Self-Care, Self-Direction, Social and Work	- It was based on the ABAS-2, and updates were incorporated to reflect the use of technologies. - It has a form for parents or caregivers, teachers, and adults. - It contains 232 items. - Its grading is manual.	- Scores were strongly correlated with the previous version of the test, in a serial clinical evaluation context [12], to verify metric equivalence.	The factorial structure of the original version is maintained
Inventory for Client and Agency Planning (ICAP) [21].	EEUU. Adapted in Chile.	From birth to over 90 years. In the Chilean study 1670 children and adolescents participated and their caregivers were the informants. [22],	- Measures the degree of support, with respect to: a) personal-social development in various areas and b) the presence and/or absence of maladaptive behaviors. - It contains 77 items.	- In Chile with confirmatory factor analysis, a 4-factor structure was obtained (Daily Life Skills, Motor Skills, Personal Life Skills, Social and Communication Skills), with adequate indices. adjustment χ2(318) = 1102.27, p < 0.001; TLI = 0.933; CFI = 0.939; RMSEA = 0.057 (IC90 %.053–0.061).	- Inventory made up of the following factors: Daily Living Skills, Motor Skills, Personal Life Skills, Social and Communication Skills
The adaptive ability performance test (ADAPT) [23]	The Netherlands	From 16 to 82 years old. Sample: 1366 clients with a possible intellectual disability seen in Dutch mental health centers.	- It is an observation instrument that is completed with the informants. - It has 65 items. - Measures conceptual, social and practical skills.	- Validity based on internal structure, through exploratory (CFI = 0.974, TLI = 0.966, RMSEA = 0.037, SRMR = 0.024) and confirmatory factor analysis (CFI = 0.907, TLI = 0.902, RMSEA = 0.067, SRMR = 0.059) and invariance measurement.	Basic self-care, hygiene and responsible eating,2. Household skills,3. Society skills, 4. Social alignment, 5. Applying school skills, 6. Dealing with money, mail, and insurance, 7. Daily structure and schedule, 8. Making responsible choices.
The functional screening tool (FST-ID) [24]	Israel	From 21 to 71 years old. Adults with intellectual disabilities. Sample: Primary care providers (N = 37), members of the multidisciplinary team (N = 8) of 92 adults aged 21 years or older, diagnosed with intellectual disability.	- It is considered a screening instrument (17 items) that requires completion by primary care service providers and a multidisciplinary team. - Offers recommendations for precise therapeutic goals, according to the results. - The application requires less than 5 min. It is accessible online.	- Validity based on content. Adherence to similar tests and review by 7 experts. - Concurrent validity: strong correlations with ABAS-II. - High values of the Alpha Coefficient (between 0.96 and 0.98) and were obtained in two groups of evaluated.	- It measures skills such as: Conceptual, Communication and academic skills, practical motor skills, independent living skills and activities of daily living, and general and challenging behaviors. The use of assistive devices and environmental change were added. A free text area was added to allow respondents to provide additional information.

Furthermore, the Adaptive Behavior Assessment System (ABAS)-3 incorporates the use of technologies such as searching for information on the internet rather than encyclopedias. This assessment has reported good test-retest reliability coefficients across all adaptive behavior domains. Nevertheless, the practical domain areas (i.e., self-care, home living, community use, health and safety, and work) showed low concordance correlations with the previous version of the scale (ABAS-2) [12]. The Inventory for Client and Agency Planning (ICAP) [21] comprises 77 items and measures motor, social, communication, personal, and daily life skills. Also, evaluate behavioral problems such as self-harm behavior and stereotyped behaviors. Recently, it has been adapted to assess children and adolescents in Chilean society [22].

Moreover, the Adaptive Ability Performance Test (ADAPT) measures adaptive functioning in adults (16–82 years old) and encompasses contemporary adaptive skills like internet banking, mobile phones, and social media; this test showed satisfactory validity and reliability [23,25]. Lastly, the Functional Screening Tool for Persons with Intellectual Disabilities (FST-ID), which is a short rapid test (17 items) with high psychometric properties [24].

In summary, currently there are few instruments that measure adaptive behavior with good psychometric properties in international samples, especially in English-speaking contexts; nevertheless, they are not freely available, distributed, or adapted to the Peruvian context. In this sense, there is a need to adapt or develop instruments culturally suitable for Peru for the measurement of adaptive behavior in people with intellectual disabilities, mainly for diagnostic and therapeutic purposes. Designing a test according to the context will favor obtaining pertinent results about personal functioning and performance. Diagnostic precision through a fair measure will avoid making wrong decisions that could impact the integral development of people with intellectual disabilities and their opportunities for socio-labor inclusion.

The construction of the Adaptive Behavior Test Battery for People with Intellectual Disabilities (ABTB-ID) will be useful to follow up on the results of intervention programs designed to improve autonomy and the development of capacities for community and labor participation in people over 15 years of age in three important areas related to social and labor inclusion. The instrument proposed in this study comprises three subscales: (a) Daily living activities, also reported in previous instruments like ICAP [22]; ABS-S:2 [19]; Vineland [13]. This subscale is essential to measuring adaptive behavior because it evaluates autonomy in meeting basic needs and how people care for themselves. The acquisition of such behaviors is important to participate in social and labor programs (ICAP [22]; ABS-S:2 [19]; Vineland [13]); more even, it allows them to be socially accepted [26]. The (b) Instrumental skills subscale includes indicators such as use of transportation, use of money, activity programming, basic reading, and the use of electronic equipment. Such skills allow the person to achieve personal objectives and goals, in addition to promoting personal autonomy and improving the quality of life [24,27]. The third (c) Communication subscale finds broad support in several instruments measuring adaptive behavior (ICAP [22]; ABS-S:2 [19]; Vineland [13]. This subscale is a challenge when working with people with intellectual disabilities [28] and is an essential condition to effectively interact with others; it is, therefore, a predictive measure of social and labor inclusion [29].

To this end, the objective of the study is to design and develop a new test battery for the assessment of adaptive behavior for people over 15 years old with intellectual disabilities, and to study its psychometric properties (i.e., to gather validity and reliability evidence).

## 2 Methodology

### 2.1 Design

This is an instrumental design including the construction and the preliminary analysis of the psychometrics properties of a measuring instrument [30]. We followed the international guidelines proposed for the test construction [31]. The process of construction and analysis of the psychometrics properties of the new instrument was achieved taking into consideration the standards test validation developed by the American Educational Research Association (AERA), the American Psychological Association (APA) and the National Council on Measurement in Education (NCME) [32].

### 2.2 Participants

#### 2.2.1 Focus group

To establish a theoretical structure that empirically reflects what we wanted to measure; we held two focus groups. The participants came from a Special Education Center in Lima-Peru. The selection criterion of the participants was intentional. A first Workshop was held with volunteers 6 fathers and 1 sister. Young people with intellectual disabilities participated in the second workshop, 5 men and 3 women who were selected by the classroom tutor.

#### 2.2.2 Cognitive interviews

Cognitive interviews were carried out to evaluate the cognitive process and interpretation of each of the items. This qualitative technique was carried out on 21 volunteer caregivers from a public special education center (No. 001- city of Tarapoto-San Martin), a region located in the northern Peruvian jungle. The 21 volunteers were mothers of adolescents with intellectual disabilities between 15 and 21 years old. 60 % of the mothers were housewives and 40 % were street traders.

#### 2.2.3 Content validity

The initial 48-item instrument was evaluated by expert judges. A total of 39 experts were selected and evaluated according to their professional skills. 22 were selected for their solid experience in psychometrics, clinical, social psychology, and specialized education. The age of the expert judges was between 29 and 62 years. 60 % were women and 40 % were men. 14 experts with bachelor's degree, 6 with master's degree, and 2 with doctorate degree.

#### 2.2.4 Reliability between observers

The participants were recruited from a Private Special Education Center in Lima-Peru. 10 adolescents with intellectual disabilities were evaluated twice with the adaptive behavior scales through their caregiver, 8 were mothers, 1 brother and 1 sister. One of the observers is a psychologist with previous experience in working with people with intellectual disabilities and the other observer is an educator with psychology studies with experience working with children and adolescents in regular basic education. They both received a training.

#### 2.2.5 Analysis of internal structure

The empirical assessment of the ABTB-ID was carried out on a sample of 566 people with intellectual disabilities from Peru. A non-probabilistic sampling procedure was carried out in two specific regions: Lima (53 %), and San Martín (47 %), located at the Coast and Jungle, respectively. The applicants were previously trained by the research group in order to ensure a standardized assessment throughout both regions. The person with an intellectual disability's caregiver is designated as an informant for the relevant questions and other sociodemographic variables. A synthesis of the sociodemographic characteristics of the sample is presented in Table 2.

**Table 2:** Sociodemographic characteristics.

Characteristics of people with intellectual disabilities		Lima		San Martin	
		Frequency	%	Frequency	%
Education level	Elementary school	169	56.3	132	49.62
	High school	106	35.3	53	19.93
	Technical studies	7	2.3	2	0.75
	University level	2	0.7	1	0.38
	Non-education	16	5.3	78	29.32
Type of family	Nuclear family	112	37.3	92	34.59
	Incomplete	92	30.7	86	32.33
	Extended family	74	24.7	80	30.08
	Reconstituted family	16	5.3	5	1.88
	Another	5	1.7	3	1.13
Sex	Male	180	60,0	142	53.38
	Female	120	40,0	124	46.62
Education system	CEBR	75	25.0	64	24.06
	CEBA	21	7.0	17	6.39
	Inclusive school	52	17.3	33	12.41
	CEBE	127	42.3	74	27.82
	CETPRO	9	3.0	1	0.38
	Non applicable	16	5.3	77	28.95
Type of Education	Public	194	64.7	177	66.54
	Private	81	27.0	12	4.51
	Total	300	100.0	77	28.95
Age	Minimum	13		13	
	Maximum	57		60	
	Media (SD)	24.92 (9.015)		30.47 (12.685)	

CEBR= Centro de educación básica regular, CEBA= Centro de educación básica alternativa, CEBE= Centro de educación especial, CETPRO= Centro tecnológico productivo

### 2.3 Measures

Following the international guidelines to design and construct a test, an observational scale was proposed to assess adaptive behavior of people with intellectual disabilities over 15 years old [31], as a screening test battery for diagnostic purposes. The 49 items in the original item pool went through several revision stages and was reduced to the final 24-item form. This test battery is composed by three sub-scales: (a) *Independence of daily living activities*, which focused on functional ability for self-care of basic activities such as eating, dressing, and cleaning up; (b) *Instrumental skills*, which explores a set of activities needed to achieve goals and objectives allowing them to be independent within society; and (c) *Communication*, which is related to the verbal and non-verbal communications, assertiveness when interacting with other people. The response format is a Likert-type ordinal scale ranging from 1 to 4; where 1 = *never*, 2 = *sometimes*, 3 = *many times* and 4 = *always*. This instrument addresses the measurement of adaptive behavior through observation and records of the main caregiver [33], in most cases a relative who knows very well the person with intellectual disability. It is advised that both, the person with intellectual disability and his caregiver are present during the assessment phase.

### 2.4 Procedure

#### 2.4.1 Construction phase

Initially, the scope and the purpose of the test was defined, then a detailed review of the scientific literature was carried out to identify the main theories and emergent dimensions of the theoretical construct as well as its nomologic network. Following the construction guidelines, we generated a matrix with the following specifications: operational definition of the construct, identification of the dimensions representing the construct, and empirically designed items. The specifications matrix was discussed by professionals with experience in working with people with disabilities, such as occupational therapists, psychologists, educational psychologists, and social workers. In this initial phase, different instruments that measure adaptive behavior were reviewed, verifying that the measurement is best carried out through observational scales.

#### 2.4.2 Item pool development

The item poll construction process followed a qualitative design under a focus group approach, as is recommended for test development [34,35]. The participants were recruited from a Special Education center and belonged to a Labor Inclusion Project. Two workshops were held, one with parents and the other with young people with intellectual disabilities, with prior explanation of the study and informed consent. The researchers encouraged the dialogue, considering the specification matrix. The workshop with the caregivers allowed the researchers to analyze and empirically validate the proposed subscales. Unanimously, the participants agreed on the multidimensionality of the construct and the need for three separate subscales for each domain. At the same time, the discussion contributed to the generation of a 48-item pool.

#### 2.4.3 Item pool review

To review the initial item pool, cognitive interviews (i.e., a qualitative technique used to obtain evidence of validity based on the response process [32,36]) were carried out in a Special Educational Center in the San Martín region with the purpose of guaranteeing the socio-cultural equivalence of each of the items. The Director of the Center made an appointment with the parents and their respective children, who were informed about the objective of the study and gave their consent for the interview. The procedure was executed using the concurrent survey technique, which consisted of asking questions about how the caregiver understands each item in addition to other open questions to find out how to culturally interpret the statements of the proposed scale [37]. Each item generated in the previous phase was critically analyzed, taking into account its clarity and comprehension. This process made it possible to refine the items, simplify them, clarify them, and eliminate those that turned out to be redundant.

#### 2.4.4 Content validity

Evidence of the content validity of the item pool was obtained through expert judgments. 39 judges were invited to a self-assessment to evaluate their expertise, which then was carried out using the k coefficient in order to filter only the most competent (details of this procedure can be found at Appendix I). The process involved giving each expert a revision form with all information related to the instrument, such as the technical sheet, specifications, and qualifications matrix. With this information, the judges assessed the clarity, coherence, and relevance of each item's content using the four-option graduated Likert-type scale [38]. Results were analyzed using the Aiken's V coefficient.

#### 2.4.5 Interobserver reliability

After establishing content validity, the item pool was tested in a small field study where 10 persons with intellectual disabilities were assessed by two trained observers, with help of the family as informants. The results of this procedure were analyzed using the Krippendorff's alpha coefficient for interobserver reliability to obtain an agreement index between observers and to verify the scores stability. This small study also helped to demonstrate that there was a clear understanding of each item by the designated caregiver.

### 2.5 Data analysis

#### 2.5.1 Content validity

As mentioned before, content validity was obtained by the Aiken's validity coefficient, which analyzes the agreement among experts. Unlike other statistics based on the agreement index between judges, this coefficient is formulated to consider all ranges of responses formulated by the judges [[39], [40], [41]]. A 95 % confidence interval was also estimated for each coefficient, obtained by using the Visual basic language, delimiting the lower limit as a threshold for content validity (95 % ≥ 0.80) [42]. Moreover, interobserver reliability was measured using Krippendorff's alpha [43,44], a statistical technique that is used to establish the test's reproducibility [45,46], and that is recommended due to its versatility in several scenarios, such as not requiring a normal distribution or complete data and is adequate when working with several observers [43,44]. For Krippendorff's alpha value, we use the available software R version 2022.02.1 + 461 (http://www.rstudio.com/) and the software irr version 0.84.1 [47]. The results of such procedure can be found at Appendix II.

#### 2.5.2 Item analysis

After the item construction and revision phases, the resulting 38-item pool was empirically tested. First, a descriptive analysis of the items was carried out mainly based on response frequencies [48] to observe each distribution and look for potential sources of bias such as outliers and the presence of potential response styles or social desirability, along with complementary analyses for analyzing multivariate normality through the Henze-Zirkler and Mardia tests expecting a non-statistically significant result [49]. The relationships between items were estimated using polychoric correlations which are suitable for ordinal variables [50] and the matrix was assessed using Kaiser-Meyer-Olkin (KMO) measure and the Bartlett's test of sphericity.

#### 2.5.3 Factor analysis

In accordance with the objectives of the study, it was decided to perform an exploratory factor analysis to determine the number of factors for each subscale and to analyze how the items are grouped with respect to the factors. For this analysis, the following steps were followed: Parallel Analysis Technique, then for the Exploratory Factor Analysis, we worked with a matrix of polychoric correlations, promax rotation and minimum residuals as an estimation method [51]. The analysis was carried out by using the JASP software ver. 0.17.3. Subsequently, the confirmatory factor analysis was performed, using the WLSMV estimator [52], because it is adequate when the inputs are ordinal variables and with a small sample size. Fit indices were obtained to confirm the new theoretical structure of the scale, using χ2 and degrees of freedom (χ2/df), root mean square error of approximation (RMSEA), residual standardized root mean square (SRMR), comparative fit index (CFI), Tucker-Lewis Index (TLI). Considering the established cut-off points: χ2/gl = values below 5 (55), RMSEA (<0.06), SRMR (<0.08), TLI (>0.95) and IFC (>0.95) [53]. Then, the degree of content homogeneity of each subscale was assessed through the Cronbach's alpha and McDonald's Omega coefficients; the latter was considered a more accurate estimation due to the congeneric measurement model underneath the subscales [54]. The analyses were carried out using the structural equation modeling (SEM) library with the JAMOVI software [55].

#### 2.5.4 Measurement invariance

As Boateng and colleagues suggest [56], at the scale evaluation phase of a development process, invariance analysis is essential to study the degree in which psychometric properties of the measurement models are transportable (i.e., generalizable) across different subpopulation groups. Nevertheless, conventional assessment of invariance under the comparison of several nested models (e.g., configural, metric, scalar, and strict) is not adequate when dealing with ordered indicators such as item responses [57]. In summary, a series of identification difficulties may be found depending on the characteristics of the assessment. For this reason, we follow a modern approach provided by Wu and Estabrook [58] to analyze measurement invariance to a degree that allows for unbiased comparisons for factor means and variances when the main inputs are ordered polytomous indicators. In this procedure, we begin by fitting a baseline model (0) and then compare it with more restricted models in which some parameters are fixed to be equal among groups. Such parameters are item thresholds (1), factor loadings (2), and intercepts (3). The analysis was coded in R, following the guidelines of Svetina and colleagues [59]. To compare the nested models, we used the incremental fit indexes ΔCFI, ΔRMSEA, and ΔSRMR in which values of ΔCFI ≤0.01, ΔRMSEA ≤0.03, and ΔSRMR ≤0.03 were delimited as thresholds to determine invariance on each level, based on popular recommendations [[60], [61], [62], [63]]. We tested for threshold, loading, and intercept invariance regarding gender, age (dichotomized based on the median), and region.

## 3 Results

### 3.1 Item descriptive analysis

**Fig. 1:**
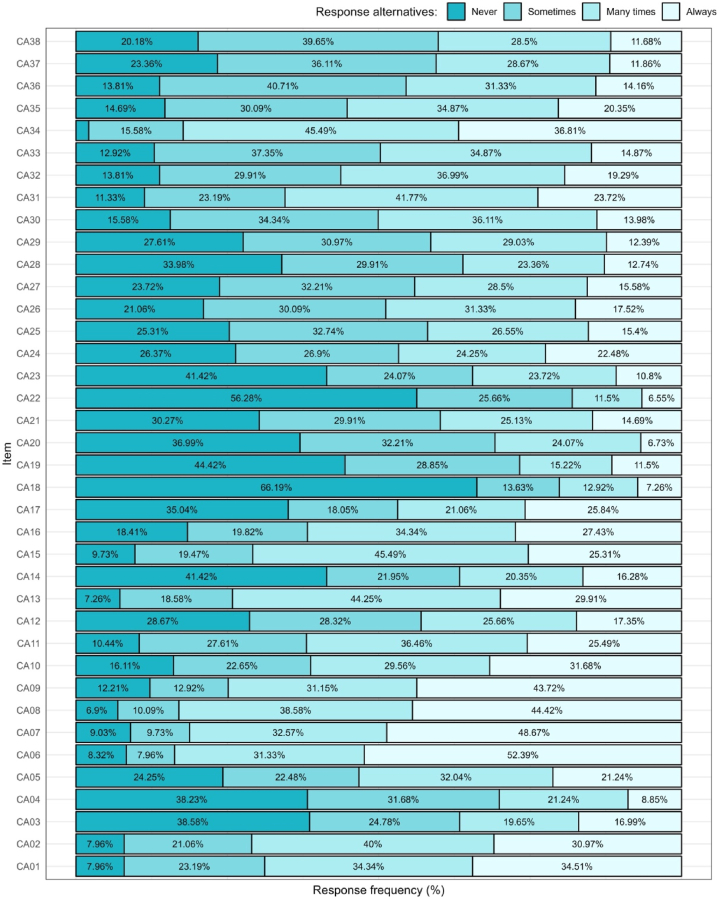
Item response frequency.

The revised 38-item pool of the scale was empirically tested. Fig. 1 shows the relative frequencies of item responses. Every item has a distinct response pattern showing that no consistent bias is present at a scale level (e.g., response styles, social desirability, floor or ceiling effects). Multivariate normality tests shown deviations from normality HZ = 1.036, p < 0.001, MSK = 17179.859, p < 0.001, MK = 55.907, p < 0.001, which supported the need for robust estimation methods. A polychoric correlation matrix was estimated in which strong relationships were observed KMO = 0.923 Bartlett's χ^2^(703) = 14971.302, p < 0.001.

### 3.2 Internal structure analysis

The polychoric correlation matrix was used as input for an Exploratory Factor Analysis. Given the characteristics of the variables, the Unweighted Least Squares (ULS) was delimited as the estimation approach, along with a promax rotation method. The main 38 items were modelled as three independent subscales and Table 3 denotes which items belong to each subscale and specific dimension within.

**Table 3:** Factorial charge by subscale.

Daily living activities	Personal Hygiene	Functional autonomy	Uniqueness	Communality
CA8	0.875		0.248	0.765
CA7	0.828		0.182	0.685
CA6	0.81		0.21	0.656
CA13	0.771		0.501	0.594
CA11	0.76		0.539	0.577
CA9	0.669		0.163	0.447
CA10	0.651		0.415	0.423
CA4		0.946	0.303	0.946
CA5		0.778	0.389	0.778
CA3		0.64	0.549	0.64
CA14		0.547	0.555	0.547
**Alpha ordinal**	0.91	0.81		
**Omega**	90	0.75		
**Instrumental skills**	**Instrumental skills**		**Uniqueness**	**Communality**
CA19	0.869		0.244	0.755
CA17	0.778		0.395	0.605
CA18	0.716		0.487	0.512
CA20	0.598		0.642	0.357
**Alpha ordinal**	0.820			
**Omega**	0.786			
**Communication**	**Verbal communication**	**Assertiveness**	**Uniqueness**	**Communality**
CA28	0.925		0.227	0.855
CA29	0.888		0.238	0.788
CA27	0.882		0.243	0.777
CA25	0.848		0.232	0.719
CA24	0.790		0.279	0.624
CA26	0.765		0.356	0.585
CA38		0.930	0.263	0.864
CA37		0.731	0.329	0.534
CA36		0.479	0.750	0.229
**Alpha ordinal**	0.931	0.744		
**Omega**	0.922	0.736		

**Daily living activities**: the results demonstrated a mean correlation between items of r = 0.304. The Parallel Analysis criterion resulted in a two-factor solution: *Personal Hygiene* items 2, 6,7,8,9,10 and 11 and *Functional Autonomy*: 3, 4 and 5. We removed the items: 1, 12, 14, 15 and 16. The explained variance was 58 %, the correlation between factors of r = 0.65 and internal consistency was ω 0.870 and 0.710 respectively.

**Instrumental Skills:** the results showed that average correlation between items was 0.329. The Parallel Analysis criterion verified one-factor structure with the following items: 17, 18, 19 and 20, in which the items: 21, 22 and 23 were removed, the explained variance was 52 % and the internal consistency of the subscale was ω 0.780.

**Communication**: the parallel analysis results detected a two-factor structure (*Verbal communication*, composed of items 24 to 33; and *Assertiveness* composed by items 34 to 38. These results demonstrated an adequate factorial solution, with an explained variance of 56 %, interfactorial correlation of r = 0.66, and internal consistency of ω 0.907 and 0.760 respectively.

Altogether, the exploratory factor analysis for each subscale provided validity evidence based on the internal structure of the subscales. To further explore the internal structure, a confirmatory factor analysis was performed for each subscale as shown in Table 4, observing adequate fit indices in the *Activities of Daily Living* subscale, CFI = 0.998, TLI = 0.997, SRMR = 0.053 and RMSEA = 0.048, the model confirmed the presence of 2 factors in accordance with the theoretical model. Furthermore, the Average Variance Extracted (AVE) was greater than 0.50, which indicates adequate discriminative power. The results of the *Communication* subscale demonstrated adequate fit, with 2 theoretically different but correlated factors. The original version proposed 3 dimensions, however, the exploratory data analysis suggested a solution of 2 theoretically justified factors. The *instrumental skills* subscale had excellent fit for a unidimensional model, as stated in its theoretical structure, however, 2 items had to be eliminated due to their low contribution, the adjustment indices were within the expected criteria: RMSEA = 0.091, X^2^/gl = 3.50, CFI = 0.989, TLI = 0.977, SRMR = 0.058, the AVE was 0.504, with an explained variance of 50 %.

**Table 4:** Adjustment indices by subscale.

Model	CFI	TLI	SRMR	RMSEA	Lower	Upper	X^2^/gl	AVE (factor 1)	AVE (factor 2)
Daily living activities	0.998	0.997	0.053	0.048	0.025	0.025	1.60	0.670	0.548
Instrumental skills	0.989	0.977	0.058	0.091	0.047	0.140	3.50	0.504	
Communication	0.995	0.994	0.051	0.073	0.055	0.091	2.63	0.664	0.547

### 3.3 Measurement invariance

Measurement invariance following modern guidelines on how to address identifiability difficulties were carried out considering threshold, loading, and intercept invariance regarding gender, age, and region. Table 5 shows the results of the analysis for the three subscales on each demographic group. In the *Daily Living Activities* subscale, we found evidence for threshold, loading, and intercept invariance in all three demographic groups, denoting that unbiased mean and variance comparisons can be estimated for the respective subscale. With respect to the Instrumental skills subscale, we found fair evidence for threshold, loading, and intercept invariance considering gender and age; nevertheless, statistically significant differences were found by examining Region; thus, denoting that some sociocultural aspects of the region may influence test results in a way that mean and variance comparisons can be slightly biased. Lastly, strong evidence for threshold, loading, and intercept invariance was found for the communication subscale in all three demographic groups.

**Table 5:** Invariance regarding gender, age, and region.

Subscale	Group	Invariance Model	CFI	ΔCFI	RMSEA	ΔRMSEA	SRMR	ΔSRMR
3Daily living activities	Gender	Baseline	0.979		0.086		0.059	
		Loadings	0.980	0.001	0.077	0.009	0.059	0.000
		Thresholds	0.981	0.001	0.072	0.005	0.059	0.000
		Intercepts	0.981	0.001	0.072	0.000	0.059	0.000
	Age	Baseline	0.984		0.080		0.054	
		Loadings	0.981	0.003	0.080	0.000	0.054	0.000
		Thresholds	0.981	0.000	0.076	0.004	0.055	0.001
		Intercepts	0.981	0.000	0.076	0.000	0.055	0.001
	Region	Baseline	0.979		0.091		0.062	
		Loadings	0.973	0.006	0.092	0.001	0.062	0.000
		Thresholds	0.974	0.001	0.087	0.005	0.063	0.001
		Intercepts	0.974	0.001	0.087	0.005	0.063	0.001
Instrumental skills	Gender	Baseline	0.986		0.105		0.041	
		Loadings	0.977	0.009	0.081	0.024	0.041	0.000
		Thresholds	0.979	0.002	0.070	0.011	0.043	0.002
		Intercepts	0.979	0.002	0.070	0.000	0.043	0.000
	Age	Baseline	0.988		0.107		0.041	
		Loadings	0.970	0.018	0.101	0.006	0.041	0.000
		Thresholds	0.967	0.003	0.094	0.007	0.049	0.008
		Intercepts	0.967	0.003	0.094	0.000	0.049	0.008
	Region	Baseline	0.989		0.099		0.039	
		Loadings	0.932	0.057	0.147	0.056	0.044	0.005
		Thresholds	0.925	0.017	0.136	0.011	0.054	0.010
		Intercepts	0.925	0.000	0.136	0.000	0.054	0.000
Communication	Gender	Baseline	0.993		0.069		0.037	
		Loadings	0.993	0.000	0.061	0.008	0.037	0.000
		Thresholds	0.993	0.000	0.056	0.005	0.039	0.002
		Intercepts	0.993	0.000	0.056	0.000	0.039	0.000
	Age	Baseline	0.993		0.072		0.039	
		Loadings	0.994	0.001	0.057	0.015	0.039	0.000
		Thresholds	0.994	0.000	0.053	0.004	0.040	0.001
		Intercepts	0.994	0.000	0.053	0.000	0.040	0.000
	Region	Baseline	0.991		0.077		0.039	
		Loadings	0.990	0.001	0.070	0.007	0.039	0.000
		Thresholds	0.991	0.001	0.065	0.005	0.039	0.000
		Intercepts	0.991	0.000	0.065	0.000	0.039	0.000

## 4 Discussion

Measuring adaptive behavior is critical to diagnosing intellectual disability [16,64]; however, several limitations in its measurement have been reported since it refers to a variety of skills that are linked to the social context [65]. In Peru, the measurement of adaptive behavior is commonly performed through commercial instruments [19,21,66] that are not accessible to most of the population; moreover, such psychometric tools were developed in international settings with distinct social and cultural characteristics that may render the test unsuitable for the Peruvian context without a proper adaptation.

For this reason, the objective of the present study was to develop a battery of tests to measure the adaptive behavior of people aged 13–60 years with intellectual disabilities, following international guidelines and standards for the construction of tests [31,32]. The starting proposal was an item pool of 49 statements, which were reduced to 38 indicators with moderate evidence of theoretical content validity and inter-observer reliability, and then empirically tested to define the final 24-item version, useful for assessment and follow-up in social-labor contexts, and also available in a mobile application for the same user.

Designing a measurement instrument is a complex process, and sometimes the authors proposing new tests do not report their approaches [67,68]. This process includes the application of a mixed methodology with structured and systematized steps. Indeed, it constitutes the foundation to ensure optimal psychometric results and is key for replication studies [56]. Theoretically, the procedures used in the construction phase of an instrument constitute the basis for the success of factor analysis, a statistical procedure that analyzes the theoretical structure of an instrument [69]. We have considered all these steps, making use of a qualitative approach, which has enormous practical utility for the test construction [34].

Another aspect to highlight is that the tests that measure adaptive behavior cover a wide age range and are made up of an excessive number of items (65 items) [23], (75 items) [67], and (117 items) [66]. Interestingly, the 24-item test battery has shown excellent psychometric properties even though its length is shorter than most proposals in the current literature.

The observer nature of the assessment is also a benefit of the scale, since one of the comorbidities associated with intellectual disabilities is difficulties in verbal communication, which makes the evaluation process more challenging [28]. Interestingly, ignoring the opinion of the person with an intellectual disability impacts the reliability of the information [70,71]. In this matter, interobserver reliability has become an important step to measure the degree of agreement or disagreement between different evaluators assessing the same thing; moreover, it is helpful to decrease observer bias and minimize subjectivity [72]. Indeed, testing reliability is an important requirement when humans are generating data from texts or observations. In this study, two observers assessed the items pool in a 5-point Likert scale and therefore, we used Krippendorff's alpha due to its versatility and flexible reliability coefficient; it can be used with different kinds of data, such as nominal, ordinal, or binary. In addition, reliability can be measured even with missing data, and most importantly, this method embraces several reliability coefficients, including Scott's π, a form of Spearman's rank correlation coefficient, and Pearson's intraclass correlation coefficient [44,73]. In addition, Krippendorff's alpha calculates disagreements among observers instead of correcting percentages agreements thus, the effect of chance is minimised [74,75]. Kreppenodrff's alpha coefficient values range between 0 (no reliability) to 1 (perfect reliability) and this value could be dependent on the number of observers, being more difficult to achieve a higher level of agreement between more observers [76].

The reliability coefficient obtained with Kripperdorff's alpha was a decision criterion for eliminating and modifying items. In summary, the values fluctuated at a modest level, making it difficult to obtain perfect agreement in the observation of behaviors of a person with disabilities [67].

Several inter-observer reliability tests have been published including the Fleiss's K, which measures the degree of agreement on observers; Cohen's kappa (k) coefficient, which could accommodate multiple observers; but it is only for nominal data, moreover one criticism has been the difficulty in interpreting the results. Lastly, Gwet, K.L (2014) [77] developed a statistical framework that embraces Bennett et al.’s S, Scott's pi, Fleiss's K and Cohen's Kappa to accommodate multiples observers, multiple observer's categories and in the presence of missing data; however, unlike the Krippendorff's alpha, this method does not minimize the effect of chance in agreement and is still controversial and need general agreement [[77], [78], [79]].

The resulting 24-item test battery was composed of three subscales: daily living activities, instrumental skills, and communication. Regarding its psychometric properties, each subscale exhibited high internal consistency of responses, which denoted reliable measures for its use in applied research. Validity evidence based on the internal structure was delimited by means of an exploratory and confirmatory factor analysis with an adequate representation of the theoretically expected dimensions. To end with, satisfactory invariance analyses demonstrated that the three subscales can be used to estimate and compare unbiased means and variances regarding gender, age, and region, with the sole exception of the instrumental skills subscale with reference to region, where it was found that there were statistically significant differences in the internal structure between participants from Lima and San Martín.

The proposed analyses are not usually carried out during the design nor adaptation of an adaptive behavior scale; even when demonstrating a robust internal structure is imperative to assert validity only a few studies report so [25]; moreover, invariance analyses are necessary in order to make fair comparisons among population groups.

### 4.1 Limitations

This instrument could be considered a self-report, and therefore, the information could be biased due to the interviewee's subjectivity. To solve this problem, stimulus presentation could be used to obtain the most objective answer.

Even when the instrument was conceptualized in a socio-laboral context, more research is necessary to verify the explanatory capacity of this scale. This is a relevant aspect of reporting evidence supporting the future expected uses of the instrument [32].

Another limitation was the accessibility of the people with intellectual disabilities, which led to a lack of randomness when selecting the people for the interview.

Poor accessibility of people with intellectual disability led to a relatively small sample size which may affect the generalization of the results in a cross-sectional study like this.

## 5 Conclusions and suggestions

In compliance with the validity and reliability requirements that a test to measure adaptive behavior must have [80], our results demonstrate that the developed scale is a valid and reliable instrument to measure adaptive behavior, including daily-living activities, instrumental skills, and functional communication [80,81].

Undoubtedly, our study could be a reference to obtain new evidence of validity considering cultural differences, gender, age, and degree of intellectual disability. A relevant aspect when providing a report of the psychometric findings of instruments that have diagnostic and therapeutic purposes is the predictive and criterion validity [32]. Consequently, the scores will allow monitoring of intervention and support programs in care centers, as well as socio-labor inclusion programs. Considering that the scale has been designed with the purpose of explaining the social and labor inclusion of people with intellectual disabilities, more studies are needed to verify the explanatory capacity of this instrument in socio-labor inclusion programs.

## Funding

The research was funded by the 10.13039/501100010747CONCYTEC (National Council for Science, Technology and Technological Innovation)-Peru. ID: PE501078008-2022.

## Ethical approval and consent to participate

The study has been approved by the Department of Psychology Research Ethics Committee (EO41-2022-03) of the University Cesar Vallejo. Written informed consent was obtained from the parents and caregivers. All methods were carried out in accordance with relevant Peruvian guidelines and regulations.

## Data availability statement

The data that support the finding of this study are openly available in zenodo.org at 10.5281/zenodo.8365539.

## CRediT authorship contribution statement

**Alicia Boluarte Carbajal:** Writing – review & editing, Writing – original draft, Visualization, Validation, Supervision, Software, Resources, Project administration, Methodology, Investigation, Funding acquisition, Formal analysis, Data curation, Conceptualization. **Gina Chávez-Ventura:** Writing – review & editing, Writing – original draft, Investigation, Conceptualization. **Jorge Cueva-Vargas:** Writing – review & editing, Writing – original draft, Methodology, Investigation, Conceptualization. **Angel Zegarra-López:** Writing – review & editing, Validation, Software, Methodology, Formal analysis, Data curation.

## Declaration of competing interest

The authors declare the following financial interests/personal relationships which may be considered as potential competing interests: Alicia Boluarte Carbajal reports financial support was provided by National Council of Science Technology and Technology Innovation.

## Figures and Tables

**Table 6:** Source to calculate the argument coefficient.

Source of argument	Influence level		
	High	Medium	Low
1. Knowledge of the thematic (national level)	0.30	0.20	0.10
2. Knowledge of the thematic (International level)	0.50	0.40	0.20
3. Experience in the field	0.05	0.05	0.05
4. Participation in related research	0.05	0.05	0.05
5. Publications	0.05	0.05	0.05
6. Continuing education	0.05	0.05	0.05
Total	1.00	0.80	0.50

**Table 7:** Knowledge coefficient (*Kc*), Argument coefficient (*Ka*) and expert competence coefficient (*K*) obtained for the experts.

Experts	Knowledge coefficient (*Kc*)	Argument coefficient (*Ka*)	Competence level (K)
1	0.3	0.8	0.55
2	0.8	1	**0.9**
3	0.7	0.8	0.75
4	0.7	0.9	0.8
5	0.9	1	0.95
6	0.7	0.8	0.75
7	0.9	1	**0.95**
8	0.9	1	**0.95**
9	0.8	1	**0.9**
10	0.6	0.8	0.7
11	0.8	0.8	0.8
12	0.5	0.6	0.55
13	0.9	1	**0.95**
14	1	0.9	**0.95**
15	0.7	0.8	0.75
16	0.8	0.5	0.65
17	0.8	1	**0.9**
18	0.9	1	**0.95**
19	0.6	1	0.8
20	0.8	0.8	0.8
21	0.7	0.8	0.75
22	0.6	0.8	0.7
23	0.9	0.8	**0.85**
24	0.9	1	**0.95**
25	0.8	0.8	0.8
26	1	1	1
27	0.9	1	**0.95**
28	0.9	0.9	**0.9**
29	0.9	0.8	**0.85**
30	0.9	0.9	**0.9**
31	0.6	0.6	0.6
32	0.9	0.9	**0.9**
33	0.8	0.8	0.8
34	0.8	0.9	**0.85**
35	0.9	0.9	**0.9**
36	0.1	0.8	0.45
37	0.6	0.8	0.7
38	0.7	0.8	0.75
39	0.8	0.8	0.8

**Table No8:** Content validity. Sub-Scale 1: Independence during daily living activities.

Items	Category	V de Aiken	IC 95 %	New items
1. Shows good manners while eating	Clarity	0.94	0.84–0.98	Two new items: 1: Remains seated while eating. 2. Chews food with the closed mouth.
	Coherence	0.96	0.87–0.99	
	Relevance	0.96	0.87–0.99	
2. Uses the spoon and fork correctly, without spilling food	Clarity	0.9	0.80–0.96	Uses the spoon and fork, without spilling the food.
	Coherence	0.96	0.87–0.99	
	Relevance	0.94	0.84–0.98	
3. Uses the knife to cut and peel food	Clarity	0.96	0.87–0.99	Idem
	Coherence	0.94	0.84–0.98	
	Relevance	0.96	0.87–0.99	
4. Prepares food without help	Clarity	0.85	0.73–0.92	Prepares food without help.
	Coherence	0.92	. 82- 0.97	
	Relevance	0.92	. 82- 0.97	
5. Serves a variety of foods without assistance.	Clarity	0.98	0.90–0.99	Serves food without help.
	Coherence	1	0.93–1	
	Relevance	1	0.93–1	
6. Handles him/herself well when he/she eats out	Clarity	0.92	. 82- 0.97	Removed
	Coherence	1	0.93–1	
	Relevance	1	0.93–1	
7. Uses the bathroom or latrine properly, without help	Clarity	0.92	. 82- 0.97	Two new items: 1. Uses the bathroom without help. 2. Cleans up his-her private parts after defecatinf or urinating
	Coherence	1	0.93–1	
	Relevance	1	0.93–1	
8. Washes her/his hands after using the bathroom or latrine	Clarity	0.94	0.84–0.98	Washes his/her hands after using the bathroom, without assistance
	Coherence	1	0.93–1	
	Relevance	1	0.93–1	
9. Bathes on his/her own initiative	Clarity	0.94	0.84–0.98	Two new items: 1. Takes a shower without helpbathes himself. 2. Takes a shower on his/her own initiative
	Coherence	1	0.93–1	
	Relevance	1	0.93–1	
10. Brushes his/her teeth correctly after eating	Clarity	0.92	. 82- 0.97	Brushes his/her teeth after every meal
	Coherence	1	0.93–1	
	Relevance	1	0.93–1	
11.Takes special care of his/her personal appearance when leaves the house	Clarity	0.98	0.90–0.99	New item: cuts his/her nails and keeps them clean
	Coherence	1	0.93–1	
	Relevance	1	0.93–1	
12. Combs his/her own hair	Clarity	1	0.93–1	Removed
	Coherence	0.98	0.90–0.99	
	Relevance	0.98	0.90–0.99	
13. Take care of his/her clothes avoiding getting dirty unnecessarily	Clarity	0.96	0.87–0.99	Keeps her/his clothes clean
	Coherence	1	0.93–1	
	Relevance	1	0.93–1	
14. Washes his/her underwear	Clarity	0.96	0.87–0.99	washes her/his clothes
	Coherence	0.98	0.90–0.99	
	Relevance	0.92	. 82- 0.97	
15. Dresses appropriately according to the context	Clarity	0.92	. 82- 0.97	Dresses according to the occasion
	Coherence	1	0.93–1	
	Relevance	1	0.93–1	
16. Sorts his/her clothes	Clarity	0.98	0.90–0.99	Idem
	Coherence	1	0.93–1	
	Relevance	0.98	0.90–0.99	
17. Wears clothes according to the weather or season	Clarity	0.96	0.87–0.99	Removed
	Coherence	0.98	0.90–0.99	
	Relevance	0.98	0.90–0.99	

Krippendorff's alpha = 0.778.

**Table No9:** Content validity. Sub-scale 2: Communication skills

Items	Category	V de Aiken	IC 95 %	New items
1. Gives correct answers about basic personal information (name, age, where she/he lives)	Clarity	0.96	0.87–0.99	Provides basic personal information (name, age, phone, address) if asked.
	Coherence	1	0.93–1	
	Relevance	1	0.93–1	
2. Relates everyday events in an understandable way	Clarity	1	0.93–1	Idem
	Coherence	1	0.93–1	
	Relevance	1	0.93–1	
3.Remembers and transmits short messages to others	Clarity	0.98	0.90–0.99	Idem
	Coherence	1	0.93–1	
	Relevance	1	0.93–1	
4. we understand what he/she says	Clarity	0.96	0.87–0.99	His language is understandable to anyone.
	Coherence	1	0.93–1	
	Relevance	0.98	0.90–0.99	
5. You can strike up a conversation with his/her	Clarity	0.9	0.80–0.96	Two new items: 1. Can start a conversation. 2. Can continue a conversation
	Coherence	1	0.93–1	
	Relevance	1	0.93–1	
6. Shows an appropriate sense of humor, depending of the situations	Clarity	1	0.93–1	Removed
	Coherence	1	0.93–1	
	Relevance	1	0.93–1	
7. Looks at the face and holds the gaze when talking	Clarity	0.98	0.90–0.99	Idem
	Coherence	0.98	0.90–0.99	
	Relevance	0.98	0.90–0.99	
8. His/her facial expression is appropriate to the context	Clarity	0.98	0.90–0.99	Removed
	Coherence	1	0.93–1	
	Relevance	0.98	0.90–0.99	
9. Maintains appropriate physical distance iduring conversations	Clarity	1	0.93–1	The item is removed, but 1 item is added: Respect personal space during the conversation.
	Coherence	1	0.93–1	
	Relevance	1	0.93–1	
10. His/her tone and volume of voice is appropriate to the situation	Clarity	0.9	0.80–0.96	Idem
	Coherence	0.92	. 82- 0.97	
	Relevance	0.92	. 82- 0.97	
11. Her/his body orientation is receptive during conversation	Clarity	0.9	0.80–0.96	Maintains straight posture during conversation.
	Coherence	0.98	0.90–0.99	
	Relevance	0.98	0.90–0.99	
12. He/she is courteous when communicating	Clarity	0.96	0.87–0.99	He/she is kind with people.
	Coherence	1	0.93–1	
	Relevance	1	0.93–1	
13. Starts a conversation by him/herself (shows initiative)	Clarity	0.98	0.90–0.99	Removed
	Coherence	1	0.93–1	
	Relevance	1	0.93–1	
14. She/he can hold a conversation for more than 5 min	Clarity	1	0.93–1	Removed
	Coherence	0.96	0.87–0.99	
	Relevance	1	0.93–1	
15. When talking, wait her/his turn to intervene	Clarity	1	0.93–1	Idem
	Coherence	1	0.93–1	
	Relevance	1	0.93–1	
16. Asks questions when she/he doesn't understand or doesn't know something	Clarity	1	0.93–1	Removed
	Coherence	1	0.93–1	
	Relevance	0.96	0.87–0.99	
17. Asks for help from others appropriately, when needed	Clarity	1	0.93–1	Three new items: 1.Controls his emotions when is angry/2.Expresses his/her disagreements when necessary/3. Accepts a contrary opinion.
	Coherence	1	0.93–1	
	Relevance	0.98	0.90–0.99	

Krippendorff's alpha = 0.674.

**Table No10:** Content validity. Sub-scale 3: Instrumental skill activities

Instrumental skills	Category	V de Aiken	IC 95 %	New items
1. Travels alone from home to another nearby place such as the store, school church	Clarity	0.94	0.84–0.98	Travels alone from home to another nearby place
	Coherence	0.98	0.90–0.99	
	Relevance	0.98	0.90–0.99	
2. Respects the traffic rules during his/her outings around the city	Clarity	0.87	0.75–0.93	Removed
	Coherence	1	0.93–1	
	Relevance	1	0.93–1	
3. Identifies and uses local transportation to get to a familiar place	Clarity	0.98	0.90–0.99	Uses local transport to get around
	Coherence	1	0.93–1	
	Relevance	1	0.93–1	
4. Knows how to gives change using coins or 10 soles bills	Clarity	0.9	0.80–0.96	Uses money to buy or pay
	Coherence	0.96	0.87–0.99	
	Relevance	0.92	. 82- 0.97	
5. Handles small amounts of money without assistance	Clarity	0.9	0.80–0.96	Removed
	Coherence	0.96	0.87–0.99	
	Relevance	0.96	0.87–0.99	
6. She/he can make a list of things she/he wants to buy. She/he knows how to spend the available money	Clarity	0.83	0.71–0.91	Removed
	Coherence	0.88	0.77 -. 94	
	Relevance	0.85	0.73–0.92	
7. Make small purchases independently at nearby grocery stores and supermarkets	Clarity	0.96	0.87–0.99	Removed
	Coherence	1	0.93–1	
	Relevance	0.98	0.90–0.99	
8. Knows the day, month, and year in which we are living	Clarity	0.94	0.84–0.98	Removed
	Coherence	1	0.93–1	
	Relevance	1	0.93–1	
9. Relates the date with his/her daily activities	Clarity	0.92	. 82- 0.97	Removed
	Coherence	1	0.93–1	
	Relevance	1	0.93–1	
10. Knows how to see and tell the time	Clarity	1	0.93–1	New item: Schedules his/her activities
	Coherence	1	0.93–1	
	Relevance	1	0.93–1	
11. Recognizes his/her written name	Clarity	0.98	0.90–0.99	Removed
	Coherence	0.98	0.90–0.99	
	Relevance	0.98	0.90–0.99	
12. Write numbers from 1 to 10	Clarity	0.94	0.84–0.98	Removed
	Coherence	0.94	0.84–0.97	
	Relevance	0.92	. 82- 0.97	
13. Writes short texts when another person dictates	Clarity	0.96	0.87–0.99	New item: 1. Acomplishes his/her activities according to his/her schedule
	Coherence	0.96	0.87–0.99	
	Relevance	0.94	0.84–0.98	
14. Reads short and simple texts (labels, labels, prices, brief instructions, brochures)	Clarity	0.98	0.90–0.99	Reads often
	Coherence	0.98	0.90–0.99	
	Relevance	1	0.93–1	
15. Uses any device or information technology (computer or cell phone or public internet booth	Clarity	0.94	0.84–0.98	Uses the computer, cell phone or tablet to search for information
	Coherence	1	0.93–1	
	Relevance	1	0.93–1	

Krippendorff's alpha = 0.528.

## Appendix I

### Expert competence assessment

Judges were invited to a self-assessment to evaluate their expertise, which then was carried out using the k coefficient. The self-assessment of the experts’ competence or K coefficient was analyzed by using the Microsoft excel (41). The experts were classified in three groups: high when K > 0.8, medium when K < 0.8 and ≥ 0.5 and low when K < 0.5 (42). We used the source of arguments indicated in Table 6.

### Expert competence coefficient (K)

We obtained the competence coefficient (*K*) of 39 promising expert judges. Our results demonstrated the presence of 19 experts with high level of competence as suggested by the k coefficient >0.8 (Table 7). They were selected in this study.

## Appendix II

### Content validity and inter-observer reliability

Results of the content validity and inter-observer reliability analyses are showed below. The items were modified or eliminated considering the lower limit of the confidence interval of the Aiken's V coefficient, the Krippendorff's Alpha, the recommendations of the expert judges, and the criteria of the researchers.

#### Daily living activities

The *functional autonomy* dimension was measured based on indicators for behaviors related to eating and dressing, the functional autonomy dimension was assessed. Aiken's validity coefficient in Food was found to be within expected cut-off points, but Krippendorff's alpha revealed moderate-to-low level coefficients. The item 6 was eliminated, and the others had their structural improvements as a result of the obtained values and the expert's advice. While in Dressing, all of the items' Aiken validity coefficients were higher than.92, indicating that the domain is adequately covered. Due to disagreements between the observers, changes to the items were considered, and item 17 was eliminated due to its resemblance to item 15 (Table 8).

Aiken's validity coefficient for the *personal hygiene* dimension is higher than.92, indicating that they have a good representation of the domain; however, due to the obtained Krippendorff's alpha, changes had to be made to a number of items. Accordingly, only item 12 was eliminated in the researcher's opinion.

#### Communication

Aiken's validity coefficient for the *verbal communication* dimension was higher than 0.80. Expert judges and observer agreements, however, recommended changing item 5 as shown in Table 9. In addition, item 6 was eliminated. The item 8 was initially removed from the nonverbal communication facet, while the items 9, 10, and 11 were modified, using the Krippendorff's alpha as the indicator.

The items for the *assertiveness* dimension were changed while taking the observer's agreement into account. As a result, item 17 was eliminated, and three additional items were suggested. The Aiken's coefficient varied between.80 and 1, indicating that the content validity was generally accurate. The reliability between observers was more variable for the Krippendorff's alpha, varying from −0.08 to.84. In this case, the researcher and the expert judges' recommendations were taken into account. Combining these findings, Table 9 shows that 7 items were changed, 5 items were eliminated, and only 2 items produced new items.

#### Instrumental skills

Following the expert judge's suggestions and Krippendorff's alpha results, we removed eight items. The remaining 7 items were modified and simplified to facilitate the comprehension of the evaluator and were evaluated as well. We have considered the following indicators: displacement and use of local transport, scheduling activities, reading, use of money, and electronic equipment.

After all the procedures were performed, 37 items were modified, and 16 items were removed. At the end, the final version consists of a 38-item instrument grouped as follows: 16 items as part of the daily-living activities, 7 items in the instrumental skills, and 15 items in the communication skills (Table 10).
